# Neural Vascular Mechanism for the Cerebral Blood Flow Autoregulation after Hemorrhagic Stroke

**DOI:** 10.1155/2017/5819514

**Published:** 2017-09-26

**Authors:** Ming Xiao, Qiang Li, Hua Feng, Le Zhang, Yujie Chen

**Affiliations:** ^1^College of Computer and Information Science, Southwest University, Chongqing, China; ^2^Department of Neurosurgery, Southwest Hospital, Third Military Medical University, Chongqing, China; ^3^College of Computer Science, Sichuan University, Chengdu, China

## Abstract

During the initial stages of hemorrhagic stroke, including intracerebral hemorrhage and subarachnoid hemorrhage, the reflex mechanisms are activated to protect cerebral perfusion, but secondary dysfunction of cerebral flow autoregulation will eventually reduce global cerebral blood flow and the delivery of metabolic substrates, leading to generalized cerebral ischemia, hypoxia, and ultimately, neuronal cell death. Cerebral blood flow is controlled by various regulatory mechanisms, including prevailing arterial pressure, intracranial pressure, arterial blood gases, neural activity, and metabolic demand. Evoked by the concept of vascular neural network, the unveiled neural vascular mechanism gains more and more attentions. Astrocyte, neuron, pericyte, endothelium, and so forth are formed as a communicate network to regulate with each other as well as the cerebral blood flow. However, the signaling molecules responsible for this communication between these new players and blood vessels are yet to be definitively confirmed. Recent evidence suggested the pivotal role of transcriptional mechanism, including but not limited to miRNA, lncRNA, exosome, and so forth, for the cerebral blood flow autoregulation. In the present review, we sought to summarize the hemodynamic changes and underline neural vascular mechanism for cerebral blood flow autoregulation in stroke-prone state and after hemorrhagic stroke and hopefully provide more systematic and innovative research interests for the pathophysiology and therapeutic strategies of hemorrhagic stroke.

## 1. Introduction

Human brain receives almost 20% of body's oxygen and glucose of cardiac output. Both oxygen and glucose are delivered to the central nervous system by cerebral blood flow (CBF) and then transported across blood-brain barrier for the brain consumption. Therefore, brain functions depend on the proper CBF due to the normal autoregulation of healthy blood vessels and cardiovascular system. If CBF stops, brain functions will shut down in seconds and neurons will be irreversibly damaged in minutes.

CBF is maintained by a coordinated action of interconnected blood vessels, which in the human brain form a 400-mile long vascular network. Within this network, cerebral arteries, arterioles, and capillaries supply the brain with oxygen, energy metabolites, and nutrients. The cerebral venous return removes carbon dioxide and metabolic waste products from the brain and into the systemic circulation for clearance. During the initial stages of hemorrhagic stroke, including intracerebral hemorrhage (ICH) and subarachnoid hemorrhage (SAH), the reflex mechanisms are activated to protect cerebral perfusion, but secondary dysfunction of cerebral flow autoregulation will eventually reduce global CBF and the delivery of metabolic substrates, leading to generalized cerebral ischemia, hypoxia, and ultimately, neuronal cell death.

**Figure 1:**
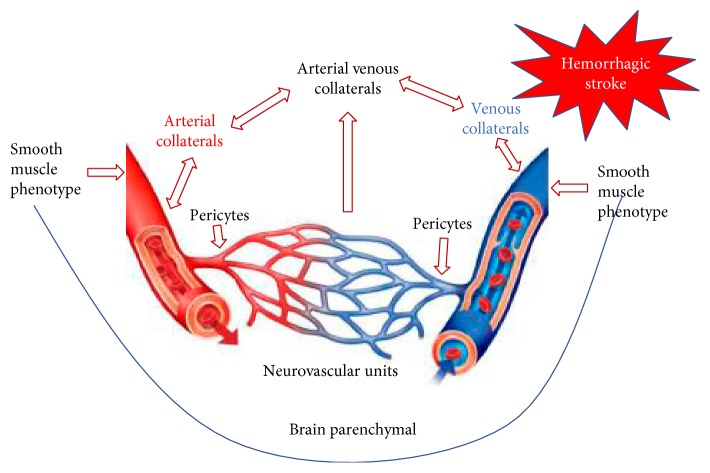
Neural vascular mechanisms for the cerebral blood flow autoregulation in the present review.

CBF is controlled by various regulatory mechanisms, including prevailing arterial pressure, intracranial pressure, arterial blood gases, neural activity, and metabolic demand. Evoked by the concept of vascular neural network, the unveiled neural vascular mechanism gains more and more attentions. This mechanism ensures a rapid increase in the rate of CBF to activated brain structures. Under physiological conditions, the capacity of increased CBF and oxygen delivery exceeds metabolic demand and oxygen consumption by activated brain sites, thus providing a large gradient for oxygen diffusion to brain cells furthest from capillaries. And different cell types, such as astrocyte, neuron, pericyte, endothelium, and so forth, are formed as a communicate network to regulate with each other as well as the cerebral blood flow. However, the signaling molecules responsible for this communication between these new players and blood vessels are yet to be definitively confirmed. Recent evidence suggested the pivotal role of transcriptional mechanism, including but not limited to miRNA, lncRNA, exosome, and so forth, for the CBF autoregulation. In the present review, we sought to summarize the hemodynamic changes and underline neural vascular mechanism for CBF autoregulation (Figure 1()) in stroke-prone state and after hemorrhagic stroke and hopefully provide more systematic and innovative research interests for the pathophysiology and therapeutic strategies of hemorrhagic stroke.

## 2. Hemodynamic Changes in Stroke-Prone State and Hemorrhagic Stroke State

### 2.1. Intracerebral Hemorrhage

ICH is the second most common cause of stroke, which initiates with brain parenchyma bleeding and hematoma growth, despite of the direct incentives [1]. Because ICH was thought to be an arterial hemorrhagic brain injury, there is little attention to the role of cerebral vein or venule in ICH pathophysiology [1, 2]. However, in the acute phase of ICH, a rapid increase of intracranial pressure due to hematoma formation could cause failure of autoregulation and reduce cerebral perfusion pressure [3]. That is why the guidelines suggest controlled lowering blood pressure treatment instead of aggressive lowering blood pressure, which intends to maintain the cerebral blood flow [4]. Moreover, recent studies found that there are new ischemic lesions coexisting with acute ICH [5–9], suggesting possible involvement of small vessel pathogenesis [5, 6].

The main secondary brain injury after ICH is thought to be three intertwined degenerative cascades adjacent to hematoma [10], including inflammation [11], red cell lysis and iron deposition [1, 12], and thrombin production [1, 12]. Moreover, besides the ischemic lesions near hematoma, there also are some remote ischemic lesions been found [3]. Similar to ischemic brain injury we reviewed above, all of these pathophysiological factors could directly and indirectly cause cerebral venule endothelial dysfunction, microthrombus, and eventually outflow reduction. Combined with other pathophysiological mechanisms, such as oxidative stress, apoptosis, and others, these factors could also lead to blood-brain barrier disruption, brain edema, and hydrocephalus, which makes further increased intracranial pressure and a vicious cycle [10]. On the other hand, most of intracranial hemorrhage occurs in hypertension patient, and the hypertensive vasculopathy, including arteries/arterioles and veins/venules, could cause “stroke-prone state” to lower the threshold of ischemic [3] and outflow dysfunction [13].

### 2.2. Subarachnoid Hemorrhage

Subarachnoid hemorrhage is a special subtype of intracranial hemorrhage, which caused by bleeding into subarachnoid hemorrhage. For a long time, cerebral vasospasm is the classic cause of delayed neurological deterioration after aneurysmal subarachnoid hemorrhage, leading to cerebral ischemia and infarction and thus to poor outcome and occasionally death [14]. However, recent clinical trials have demonstrated marked prevention of vasospasm with the endothelin receptor antagonist clazosentan, yet patient outcome was not improved [15, 16]. These disappointing results reminded researchers switching interests into early brain injury [17, 18], but this concept is merely limited in neurons and overlooks the functions of other cell types. Fortunately, recent evolving concepts, such as neurovascular unit [19], vascular neural network [20], and then vasculo-neuronal-glia triad model [21], noticed the contributions of cerebral microcirculation. However, they all keep cerebral veins and venules at an arm's length.

Rethinking of the failed clazosentan clinical trials, there might be a missing factor that, compare to arteries, endothelin only has less potent as a constrictor in cerebral veins [22], which means powerful endothelin receptor antagonist clazonsentan may not alleviate the “vasospasm” in cerebral venous system after subarachnoid hemorrhage. Moreover, clazosentan did not prevent the formation of microthrombi [23]. Recent studies found that there is also vasospasm in deep cerebral veins after subarachnoid hemorrhage [24], and the diameter significantly decreased 1 day and peaked at 5–7 days after subarachnoid hemorrhage [25]. In the meantime, whether there has diameter reducing in cerebral venules after SAH is still controversial [26–29]. In addition, SAH elicited time- and size-dependent increases in rolling and adherent platelets and leukocytes in cerebral venules [30], which lead to microthrombus and microvascular stasis [29, 31]. Similar to other brain injuries, subarachnoid hemorrhage can also cause brain edema [21, 32, 33], hydrocephalus [34, 35], and then cerebral hypoperfusion [36] just like we reviewed above.

In another way, cerebral venous thrombosis [37–40] or stenosis [41] is also an uncommon etiology of subarachnoid hemorrhage, mostly perimesencephalic subarachnoid hemorrhage [42–44]. Potential cause may be elevated intracranial venous pressure or mechanical swelling of the intracranial venous system, leading to variant of cerebral venous drainage [45–49], arteriovenous malformation [50], and eventually veins or venule breakdown [36, 38, 51].

### 2.3. Hypertension

Hypertension is one of the most important risk factors of brain injuries. Sustained high blood pressure could cause smooth muscle cell hypertrophy and then vessel remodeling [52], eventually leading to vessel lumen stenosis and decreased venous distensibility [53, 54]. In the meantime, hypertension could increase the collagen biosynthesis and deposition in perivascular spaces [55], which could have similar effect to the perivenous cuffs in MS patients [56]. However, the major vascular complication under hypertension condition is endothelial dysfunction [57], which will lead to BBB disruption [58–61] and impairment of vascular tone modulation [57]. In addition, hypertension could also cause adherent leukocytes and platelets in cerebral venules [62]. All these pathophysiological effects could increase cerebral venous pressure, impair cerebral venous outflow [63–65], and eventually rCBF reduction [66].

### 2.4. Diabetes

Diabetes is another major risk factor of stroke. Diabetes develops because of inadequate pancreas islet *β*-cell and adipose-tissue responses to chronic fuel excess, which results in nutrition excess, insulin resistance, and metabolic stress [67]. Among these, metabolic stress leads to endothelial dysfunction, including cerebral venous system, which is considered to be the initial process in vascular manifestations of diabetes [68, 69]. Following vascular related alterations involves platelet adhesiveness and coagulation cascade, vasoconstriction, and inflammation [69, 70]. Similar to other brain injuries, these pathophysiological changes ultimately lead to cerebral venous thrombus [71, 72], venous hyperaemia, and brain edema [73].

## 3. Neurovascular Networks as Future Therapeutic Targets

### 3.1. Pericytes as a Potential Interventional Target

Pericytes cover venules of superficial cerebral veins in the central nervous system as well as arterioles, which determine the contraction and dilation of these vessels. Recent evidence has suggested that pericytes secrete matrix metalloproteinase-9 to degrade the endothelial matrix and blood-brain barrier around their somatic bodies. In addition to mechanic stress caused by hyperperfusion after recanalization [74], pericytes may have a fundamental role in the disruption of the blood-brain barrier in poststroke venules but not arterioles and capillaries. This detrimental function may have been induced by cyclophilin A and its downstream signaling pathways.

However, pericytes have multipotential functions that could underlie blood-brain barrier development and repair. First, pericytes can form intercellular tight junctions in the blood-brain barrier [75]. Additionally, pericytes also contribute to the formation of the basal lamina by synthesizing type IV collagen, glycosaminoglycans, and laminin (Allt and Lawrenson, 2001). Large efforts have been undertaken to induce angiogenesis and protect the blood-brain barrier [76–79], but it is still far from clinical application. Recently, we successfully stimulated tight junction and adherens junction proteins by activating Frizzled-4 receptor, a canonical Wnt signaling receptor that is also expressed on pericytes [80], suggesting that pericytes may be a promising target to maintain blood-brain barrier integrity and functions during subarachnoid hemorrhage treatment [21].

Furthermore, previous evidence suggests that the occurrence of global ischemia after subarachnoid hemorrhage significantly constricts pericytes and reduces blood flow in the microcirculation. However, in contrast to intuition, dilated pericytes may not reverse blood flow, which we called a “no-reflow phenomenon” [81, 82]. Second, during subarachnoid hemorrhage and other brain injury pathophysiologies, pericyte contraction, usually together with pericyte-programmed cell death, caused a reduced capillary density and maintained an inactive microcirculation [27, 83, 84]. Our recent studies indicated that pericytes are induced to contract in response to hemoglobin and nitric oxide/cGMP pathway, forming pearl string-like contractions in microvessels to deteriorate the microcirculation [85].

In addition, pericytes could modulate the proliferation, migration, and differentiation of endothelial cells; pericytes cocultured with endothelial cells and astrocytes could establish a stable capillary-like structure [86, 87]. In their efforts to orchestrate initiation, sprout connection, and termination in angiogenesis, pericytes secrete vascular endothelial growth factor and interleukin-6 to facilitate endothelial cell maturation and microvessel sprouting, contributing a pivotal role in the initial stage of angiogenesis [88]. Transforming growth factor-*β* binds to its receptor on endothelial cells and pericytes to self-regulate and induce perivascular mesenchymal cell differentiation into pericytes and smooth muscle cells [89]. Moreover, the platelet-derived growth factor (PDGF) pathway is the crucial factor for sprouting capillary recruitment of pericytes. Angiopoietin-1 from pericytes binds to Tie-2 on the endothelial cell to enhance pericyte surrounding of the new blood spout, increasing the vessel stability [90].

Due to their specific spatial distribution along microvessels and their broad cellular properties, pericytes could be an ideal target for the development of novel preventive and therapeutic strategies by modulating and controlling the neural vascular network, consequently improving neuroprotection [91]. For example, targeting pericytes during the development of microvascular dysfunction and elucidating the molecular pathways involved in the regulation of pericyte activities for attenuating chronic rejection intervention have been demonstrated [92]. A recent review also targeted pericytes as clinical endpoints and therapeutic interventions in diabetic retinopathy [93]. Interestingly, maintaining high levels of estrogen E2 are critical for the control of PDGF-mediated crosstalk between endothelial cells and pericytes, which governs the microvessel stability and is essential for preserving intracranial homeostasis, consequently reducing the risk of intracranial hemorrhage and decreasing the incidence of stroke and cerebral aneurysm [94]. Future studies should further determine the role of pericytes before and after hemorrhage to illustrate the mechanism underlying the occurrence and development of this critical disorder. Additional drugs and trials targeting pericytes and their effectiveness are also required to develop new strategies for the prevention and treatment of hemorrhagic stroke.

### 3.2. Smooth Muscle Phenotype for Autoregulation

In addition to pericytes, smooth muscle cells also contribute to regulation of the cerebral blood supply with much greater strength. In fact, some researchers consider the smooth muscle cells as the dominator for microvessel autoregulation. In 1993, Contard et al. demonstrated that smooth muscle phenotypes in stroke-prone spontaneously hypertensive rats had no effect on blood pressure or associations with thickness [95]. Most importantly, the changes in the smooth muscle cell phenotype may be beneficial for ischemic tissue lesions in the heart [95]. After traumatic brain injury, mechanical stress can also induce subarachnoid hemorrhage, similar to vasospasm in response to smooth muscle cell hypercontractility and phenotype switching for prolonged vessel remodeling and lumen occlusion. Our recent experiments also propose a potential role for the maintenance of the cerebral smooth muscle phenotype in early brain injury after subarachnoid hemorrhage [96].

Regarding its internal mechanism, the smooth muscle phenotype was mainly regulated by platelet-derived growth factor-BB (PDGF-BB), which has been reported to stimulate smooth muscle cell differentiation, proliferation, and phenotypic transformation [97]. Additionally, PDGF-BB induces the differentiation of the bone marrow endothelial progenitor cell-derived cell line TR-BME2 into mural cells/pericytes and alters the smooth muscle cell phenotype [98]. Other studies have suggested that the ACTA2 gene, calcium signals, cadherin 6B, and integrin receptor may also participate in this pathophysiological process [96, 99–101]. Due to limited evidence in this field, especially in the central nervous system, additional efforts are still needed to elucidate the pivotal role of the smooth muscle phenotype in autoregulation after stroke and other central nervous system disorders.

### 3.3. Collaterals for Recirculation

Due to the great contributions of the collateral circulation to stroke outcomes, we wondered how to manipulate this important and neglected factor in previous stroke pathophysiologies and therapies. Current strategies include the following. (1) Statins may open collaterals after stroke, preserve penumbra, and expand the time window of thrombolysis [102, 103]. Ovbiagele et al. evaluated the relationship between prestroke statin use and pretreatment angiographic collateral grade among patients with acute ischemic stroke, and they found that the statin-treated group had significantly higher collateral scores than the nonstatin users, suggesting an association between statin use and improved collateralization during acute stroke [104]. (2) Intracellular chloride channel 4 is a determinant of native collateral formation in the brain [105]. Chalothorn et al. observed reduced collateral formation in mice that were deficient in chloride intracellular channel 4, which displayed greater ischemia and worse perfusion [106] and recovery [106]. (3) Vascular endothelial growth factor (VEGF) may be specific for collateral development. Harrigan et al. treated MCAO rats with chronic intraventricular infusions of VEGF, which increased the vascular density in a dose-dependent manner and minimized the associated brain edema after ischemic stroke [107, 108]. (4) Pioglitazone reduces the nonflow phenomenon in microvessels. Shimazu et al. found that the peroxisome proliferator-activated receptor-gamma (PPAR*γ*) agonist reduces the infarction size in transient but not permanent MCAO, suggesting that the role of PPAR*γ* is specific to events that occur during reperfusion, possibly the collateral circulation [109]. During the postischemic, reperfusion phase, pioglitazone, a synthetic agonist for PPAR*γ*, also improves recovery from ischemic stroke [110]. Nevertheless, due to the outlook for collateral circulation, especially the venous collaterals, in translational stroke research over the past decades, limited strategies have been discovered and developed. In future studies, an improved understanding of collateral hemostasis after stroke and of precision therapeutic therapies is highly encouraged. And the pathophysiological therapeutic time window, depending on the collateral circulation of the patient, might replace the current suggested time window for the endovascular treatment after stroke.

## 4. Transcriptional Signals for the Autoregulation

### 4.1. Classical Molecule Signals

As we summarized above, cerebral autoregulation, an inherent ability to maintain a relatively steady-state CBF despite fluctuation in arterial blood pressure, is attributed to an intrinsic ability of smooth muscle cells and pericytes to constrict or relax to minimize variation in CBF. The signaling event underlying myogenic response consists of an activation of stretch-activated Ca^2+^ channels by an increase in intravascular pressure [111]. This results in an elevation in intracellular Ca^2+^ and subsequent stimulation of phospholipase A2, leading to the release of arachidonic acid from membrane phospholipids. Arachidonic acid metabolites, 20-HETEs, inhibit Ca^2+^-dependent K channels resulting in depolarization of smooth muscles and vasoconstriction. Functional hyperemia implies an increase in CBF induced by neural activity to meet the local metabolic demand. This is a well-coordinated event involving neurons, astrocytes, and vascular cells.

It is widely assumed that calcium-dependent release of vasoactive substances by astrocytes results in arteriole dilation and the increased blood flow which accompanies neuronal activity. Howarth [112] summarized the evidence which has convincingly demonstrated that astrocytes are able to modify the diameter of cerebral arterioles. Howarth discussed the prevalence, presence, and timing of stimulus-induced astrocyte calcium transients and described the evidence for and against the role of calcium-dependent formation and release of vasoactive substances by astrocytes.

### 4.2. Transcriptional Modulators

Nuclear factor-kappa B (NF-kappaB) is a multisubunit transcription factor that when activated induces the expression of genes encoding acute-phase proteins, cell adhesion molecules, cell surface receptors, and cytokines. Stephenson et al. [113] demonstrate that transient focal cerebral ischemia results in activation of NF-kappaB in neurons and supports previous observations that neuroprotective antioxidants may inhibit neuronal death by preventing the activation of NF-kappaB. Samraj et al. [114], using system biology tools and experimental SAH models, have identified signal transducer and activator of transcription 3 (STAT3) transcription factor as a possible major regulatory molecule in late cerebral ischemia after subarachnoid hemorrhage.

### 4.3. Genomic Targets for Autoregulation

miRNAs play important regulatory roles in a variety of cellular functions as well as in several diseases, including stroke. Jeyaseelan et al. [115] showed miR-103 and rno-miR-107 related to transient focal ischemia by middle cerebral artery occlusion. Wang et al. [116] concluded that miR-29b could potentially predict stroke outcomes as a novel circulating biomarker and miR-29b overexpression reduced BBB disruption after ischemic stroke. MicroRNA-210 (miR-210), a master and pleiotropic hypoxia-microRNA, plays multiple roles in brain ischemia. Zeng et al. [117] valuate the correlation of blood miR-210 with clinical findings in acute ischemic stroke and found blood miR-210 is a novel sensitive biomarker for clinical diagnosis and prognosis in acute cerebral ischemia. Yin et al. [118] suggest that miR-497 promotes ischemic neuronal death by negatively regulating antiapoptotic proteins, bcl-2 and bcl-w. We raise the possibility that this pathway may contribute to the pathogenesis of the ischemic brain injury in stroke. Gan et al. [119] demonstrated that hemostatic mechanisms are affected by ischemic stroke and concluded that circulating microRNA-145 has potential as a biomarker for ischemic stroke.

### 4.4. Big Data Analysis for the CBF Autoregulation

Many data mining methods are used in the field of CBF regulation, such as nonlinear analysis [1], which is often used to analyze the relationship between CBF regulation and other factors. For example, Saleem et al. [120] determine the consistency of dynamic cerebral autoregulation by characterizing the pressure-flow relationships. Mitsis et al. [121] build a nonlinear model of the dynamic effects of arterial pressure and blood gas variations on cerebral blood flow in healthy humans. Tan [122] defined the characteristic relationship between arterial pressure and cerebral flow. Mitsis et al. [123] assessed by examining the dynamic relationship between spontaneous fluctuations of cerebral blood flow and arterial blood pressure under various levels of lower body negative pressure in healthy humans. Other data mining methods are used in the field too. Chiu et al. [124] use time domain cross-correlation analysis of prefiltered mean arterial blood pressure and mean cerebral blood flow velocity which were applied to assess the cerebral autoregulation. Liau et al. [125] use time domain cross-correlation function which was applied to evaluate the relationship between blood pressure and cerebral blood flow velocity signals acquiring from healthy subjects and stroke patients both in supine and head-up tilt positions to evaluate the effect of posture change. Chacón et al. [126] posit a nonlinear model of the CBF autoregulation system through the evaluation of various types of neural networks [127] that have been used in the field of system identification. Chiu et al. [128] use support vector machine to [129] build a classification of dynamic cerebral autoregulation in diabetics with autonomic neuropathy. Liau et al. [130] used chaotic analysis [131] in diabetic autonomic neuropathy and assessed dynamic cerebral autoregulation and suggested that impaired autoregulation would be more chaotic and less predictable.

Although many data mining techniques have been applied to the field, but in some researches, the amount of data is small, which has a great impact on the accuracy of the algorithm or model. At present, big data [132] are very broadly used, and we can use big data technology to improve this problem. There are a lot of big data applications on the field of stroke therapy [133–135], but there is little reference to big data in the cerebral blood flow autoregulation research. There are numerous imaging techniques such as SPECT, CT, MRI, and PET used in the cerebral blood flow research field [136], and as we know, the amount of image data is far more than the other data, and we can use big data technology and its 3V (velocity, volumes, and variety) [132] feature to improve the algorithm or model. Big data can also be used for genetic data analysis to find genomic targets for autoregulation. Also, the computer industry has transitioned into multicore and many-core parallel systems [137] and GPU programming like CUDA [138] are wildly used in speeding up algorithms. We can also employ high performance computing and related data mining algorithm [139–145] to speed up the algorithms of the cerebral blood flow research.

## 5. Hemodynamic Changes for Therapeutic Strategies of Hemorrhagic Stroke

The central spirit of summarizing CBF autoregulation mechanism is to help reperfusing the ischemic brain region after hemorrhagic stroke. Compared to the progressive stage of shock, when the compensatory mechanisms begin to fail, blood remains in the capillaries, leading to tissue anoxia, and there are somehow similarity with cerebral congestion under cerebral venous dysfunction. We might get benefit to diagnose the prevalence of cerebral venous dysfunction by monitoring rCBF [146, 147] due to autoregulation failure and blood congestion, SjvO_2_ [148, 149] due to significantly reduced brain energy consumption, and lactate [150, 151] of internal jugular vein due to sustained anaerobic metabolism in related brain regions. Moreover, carefully monitoring cerebral venous drainage could be applied to prognostic evaluation after brain injury [152].

Current reperfusion treatment barely involves cerebral venous system, including surgical interventions such as aneurysm clipping or coiling [153], which can be used to prevent rebleeding after subarachnoid hemorrhage. Mechanical clot-retrieving devices [154] or chemical agents such as rtPA [155] are used to reopen occluded arteries. However, these treatments might not effectively restore the blood flow in capillary and downstream venous system. Existing data support the use of systemic anticoagulation as an initial therapy in all patients [156], even in the presence of hemorrhage. Interestingly, Simard et al. recently demonstrated that low-dose intravenous heparin infusion after surgery in patients with aneurysmal subarachnoid hemorrhage is safe and beneficial [157]. Furthermore, while controversial, surgical interventions are being used to reverse the possible pathogenesis chronic cerebrospinal venous insufficiency [158]. And improved decompressive craniotomy could alleviate intervention toward cerebral venous system and less brain damage [36, 159]. In sum, carefully monitoring and treating the cerebral venous dysfunction are critical, therefore, to effectively restore optimal cerebrovascular function.

## 6. Perspective and Conclusion

During the initial stages of hemorrhagic stroke, including intracerebral hemorrhage and subarachnoid hemorrhage, the reflex mechanisms are activated to protect cerebral perfusion, but secondary dysfunction of cerebral flow autoregulation will eventually reduce global cerebral blood flow and the delivery of metabolic substrates, leading to generalized cerebral ischemia, hypoxia, and ultimately, neuronal cell death. Evoked by the concept of vascular neural network, the unveiled neural vascular mechanism gains more and more attentions. Different cell types and molecular and transcriptional modulators may be involved in the neural vascular mechanism for CBF autoregulation. However, current understandings could not explain all the clinical phenomenon and strategies for autoregulation after hemorrhagic stroke.

Recently, application of the cerebral venous dysfunction for hemorrhagic stroke pathophysiology presents an opportunity to identify how cerebral venous system is involved in the prone vulnerability of brain injury and the control of reperfusion. This strategy expands the vascular neural network by improved understanding of cerebral venous system playing a key role in the mechanism of brain injury. However, more research is needed to figure out the time course of cerebral venous changes after hemorrhagic stroke and their implications for the CBF autoregulation. And studies are also needed to investigate the interactions between different kinds of cell types in the CBF autoregulation before and after hemorrhagic stroke. Communications among venous endothelial cells, pericytes, astrocytes, smooth muscle cells, and perivascular neurons should be studied systematically to elucidate how and when these happen. More precised animal models and detective method toward CBF autoregulation are also needed. Ultimately, these efforts should facilitate the development of therapeutic strategies, no matter surgeries or pharmacological agents target the sewerage system of the brain and all kinds of plumbers that serve to build, maintain, and regulate it.
